# Agent-based modeling of the interaction between CD8^+^ T cells and Beta cells in type 1 diabetes

**DOI:** 10.1371/journal.pone.0190349

**Published:** 2018-01-10

**Authors:** Mustafa Cagdas Ozturk, Qian Xu, Ali Cinar

**Affiliations:** 1 Department of Chemical and Biological Engineering, Illinois Institute of Technology, Chicago, IL, United States of America; 2 Department of Biomedical Engineering, Illinois Institute of Technology, Chicago, IL, United States of America; Baylor College of Medicine, UNITED STATES

## Abstract

We propose an agent-based model for the simulation of the autoimmune response in T1D. The model incorporates cell behavior from various rules derived from the current literature and is implemented on a high-performance computing system, which enables the simulation of a significant portion of the islets in the mouse pancreas. Simulation results indicate that the model is able to capture the trends that emerge during the progression of the autoimmunity. The multi-scale nature of the model enables definition of rules or equations that govern cellular or sub-cellular level phenomena and observation of the outcomes at the tissue scale. It is expected that such a model would facilitate *in vivo* clinical studies through rapid testing of hypotheses and planning of future experiments by providing insight into disease progression at different scales, some of which may not be obtained easily in clinical studies. Furthermore, the modular structure of the model simplifies tasks such as the addition of new cell types, and the definition or modification of different behaviors of the environment and the cells with ease.

## Introduction

Type 1 diabetes (T1D) is an autoimmune disease, in which the insulin-producing Beta cells in the pancreas are destroyed by the immune system, typically leading to complete insulin deficiency [1]. Although T1D is considered to constitute 5–10% of all cases of diabetes [2], its incidence was reported to have increased significantly in the past few decades [3], especially in children under five [4]. While there has been continuous efforts toward the elucidation of the biological mechanisms involved in disease pathogenesis and the optimization of treatment options, the required resources and time for the clinical testing limit the number of studies.

Computational modeling is a powerful tool for assessing the feasibility of potential interventions and therapies, as well as hypothesis testing. *In silico* experiments can be performed quickly and cost-effectively under a wide variety of conditions, and the results can be used to plan *in vivo* or *in vitro* clinical studies. Depending on the structure of the model, it is also possible to investigate the causality between certain events or behavior of certain components within the system.

Many models with specific goals have been proposed for T1D, and recent reviews were provided by Ajmera et al. [5], and Jaberi-Douraki et al. [6]. While the majority of modeling efforts focus on glucose-insulin homeostasis, a number of studies focus on modeling the autoimmune response in T1D. Freiesleben De Blasio et al. [7] proposed an ordinary differential equation (ODE) based model, commonly known as the *Copenhagen model*, which depicted the interplay between T cells, macrophages and antigens. Although the goal of this model was to predict T1D qualitatively through stability analysis, it was revised later by Marée et al. [8] for quantitative predictions on non-obese diabetic (NOD) and control (Balb/c) mice. Later, Shoda et al. [9] developed a highly complex model of T1D in NOD mice, PhysioLab^®^, based on algebraic and ordinary differential equations representing different compartments in the body. This detailed model, which included over 300 ODEs and over 1000 parameters and algebraic equations, was mainly aimed towards drug candidate trials [9]. Additionally, there have been mathematical modeling studies that focused on immune cell populations [10–12] and immune cell cycles [13]. Lastly, there have been modeling efforts that utilized other methods such as agent-based modeling (ABM) to study T1D [14, 15]. These studies were geared toward studying the interplay between macrophages and Beta cells, and did not include T cells. More recently, Wedgwood et al. [16] developed an agent-based model of human insulitis that included T cells and B cells in addition to the Beta cells. The model considered a single islet composed of Beta cells and the islet basement membrane, and investigated the time course of insulitis progression by varying model parameters such as B and T cell counts in the inflammatory infiltrate.

The agent-based model we present considers NOD mouse insulitis in a tissue section composed of islets of varying sizes, CD8+ T cells with naive, effector and memory functions, and Beta cell regeneration. The model is composed of components such as simulation space(s), different agents representing different cell types, literature-derived rules that define cell-cell and cell-environment interactions, and parameter values specific to each component. Such a modular framework makes tasks such as the expansion of the model by addition of new cell types, or modification/introduction of new rules based on recent literature, a straightforward task that does not compromise model integrity. Mainly, the proposed model has two distinguishing features. First, the model provides mechanistic insight into the progression of T1D and dynamic prediction of the spread of inflammation. Insulitis is a spatially heterogeneous process which can be effectively replicated by an agent-based approach. Secondly, the model was built on a high-performance computing (HPC) framework, which enables simulations ranging from a small section of pancreatic tissue that can be performed on a personal computer to a significant portion of the mouse pancreas involving several millions of cells that can be carried out on a supercomputer. The model, which operates at a moderate complexity, aims to provide predictions that are accurate enough to guide clinicians in planning of experiments and hypothesis testing with reduced cost and time, in addition to allowing the modification, expansion and calibration against clinical data.

In the following sections, we first give an introduction to ABM, a versatile tool for multi-scale modeling. Next, we describe the model structure, as well as the components used in the model with pertinent references. In Section 3, we present simulation results under a variety of conditions and discuss the findings. Finally, we conclude our findings in Section 4 and discuss future research directions.

### Agent-based modeling

Most living systems are made up of several components and subsystems (organs, cells, organelles, etc.), the number of which can vary from just a few to several trillions [17]. Various types of interactions between these components lead to the complex behavior of the overall system that is usually observed at a higher spatial and/or temporal scale than that of the components. This complex behavior is more precisely referred to as the *emergent behavior* within the scope of complexity science, which often cannot be inferred by merely analyzing individual components.

Agent-based modeling is a modeling paradigm where the components of the system of interest are represented by *agents*—autonomous software fragments. Similar to the actual system, the agents can interact with the environment and other agents to replicate the emergent behavior. The advantages of multi-scale ABM include modularity and the ability to observe events at multiple scales (*e.g*. cellular or tissue level), which elucidates potential pathways leading to the emergent behavior. Based on whether the emergent behavior is one that is to be avoided (such as the autoimmune response in T1D), or one that is preferred, potential interventions to the system can be devised to achieve the desired outcome.

Use of ABM has expanded in the last few decades, and it has become a popular tool in many disciplines including social sciences, business, economics, technology and network theory. More recently, ABM gained popularity in biological sciences. Some examples of application include bone tissue engineering [18, 19], angiogenesis [20–23], breast cancer research [24, 25], and immune system modeling [26–28].

## Model structure and components

The model was developed on the Repast High-Performance Computing (HPC) toolkit [29], which provides a convenient parallel programming environment to develop ABMs. This allows the model to be run on supercomputers, potentially enabling the simulation of the whole mouse pancreas (∼3000 islets [30, 31]), or a significant portion of the human pancreas (∼3.2 million islets [32]).

The results presented in the following sections were obtained by simulating a 200 × 200 grid 2-dimensional (2D) tissue section, where each grid location represents a 10 × 10 *μm* tissue section. Although the model represents a slice of the actual pancreatic tissue, we assume that the interactions within the vicinity of the simulated slice would be very similar and therefore expect the 2-dimensional simulation outcomes to be fairly close to those that would be obtained from a 3D representation. Also, the time resolution of the model was chosen to be 1 minute, which allows sufficient time for cellular and sub-cellular event dynamics to evolve.

The simulations were conducted considering non-obese diabetic (NOD) mice, one of the widely-used animal models [33, 34]. For NOD mice at approximately three weeks of age, dendritic cells (DCs) and macrophages are reported to infiltrate the pancreatic tissue, followed shortly by the recruitment of CD8^+^ T cells [33, 35]. Since the model focuses on the direct interaction between CD8^+^ T cells and the islets, the simulations were assumed to start at 4 weeks of age and lasted for 10 weeks (14 weeks of age) [1, 36]. It is assumed that, by 4 weeks of age, the initiation of immune response by the DCs and macrophages has reached a level where CD8^+^ T cells are present in the pancreatic tissue, and that the majority of interactions take place between the islets and the CD8^+^ T cells. A general description of T cell/Beta cell interactions and governing rules can be found in S1 Fig.

### *CD*8^+^ cells

Insulitis occurs as a result of the recruitment of *CD*4^+^ and *CD*8^+^ T cells from the peripheral circulation, as well as the proliferation of already recruited cells [37]. During this process, naive *CD*8^+^ T cells differentiate into effector T cells (*T*_*eff*_), which can then kill Beta cells through the perforin-granzyme pathway [38–40].

There are 3 subsets of *CD*8^+^ T cells considered in our work: naive, effector and memory *CD*8^+^ T cells. In the simulations, the effector *CD*8^+^ T cells are produced either through differentiation from naive T cells or proliferation of effector T cells. This is based on the activation of naive *CD*8^+^ T cells through antigen presentation by islet cells (via major histocompatibility complex (MHC) class I), or by other immune cells such as dendritic cells and macrophages (via MHC class II) [41]. Here we assume that the activation of naive *CD*8^+^ T cells (which is known to take place in lymph nodes by antigen presenting cells) can also happen in the pancreatic tissue based on recent clinical studies [38]. Therefore, we assume that T cells can recognize Beta cells and act (i.e. become activated, engage, or differentiate) accordingly, based upon contact of the T cell receptor (TCR) and the MHC class I, which is typically located on the surface of Beta cells [6]. Similarly, memory T cells are produced through differentiation from effector T cell parents. The memory T cells can acquire effector function upon coming into contact with their cognate antigen.

The migration rate of *CD*8^+^ T cells into the pancreas from the pancreatic lymph node (PLN) was taken as 1.7% per day based on the population dynamics study of islet infiltrating cells carried out by Magnuson et al. [38]. Furthermore, intravital two-photon imaging studies conducted on mice demonstrated that the T cells moved autonomously and independently within the pancreatic tissue, suggesting a random walk behavior rather than collective migration induced by chemotactic gradients [42, 43]. In the light of these studies, the movement of *CD*8^+^ T cells was modeled as random walk with a persistence time of 1 to 4 minutes and a movement speed of 10 *μm*/*min* [43].

The lifespan of naive T cells was chosen to be 8 weeks [44]. Although there does not seem to be a consensus on the lifespan of effector *CD*8^+^ T cells, most studies on mice and humans suggest a lifespan of 5–8 days [45, 46]. Therefore, the lifespan of effector *CD*8^+^ T cells was chosen to be 6 days. In the case of memory *CD*8^+^ T cells, the lifespan was reported to be between 6 months to 1 year in mice [47] and was set to be 6 months in the simulations.

Although the different subsets of T cells in the simulation have different lifetimes, all T cell types can disappear from the simulation by moving beyond the boundaries of the simulated tissue, in which case they are assumed to have migrated to the neighboring tissue sections. Similarly, new T cells can appear near the boundaries to mimic the incoming migration of T cells from the surrounding tissue sections.

**Fig 1:**
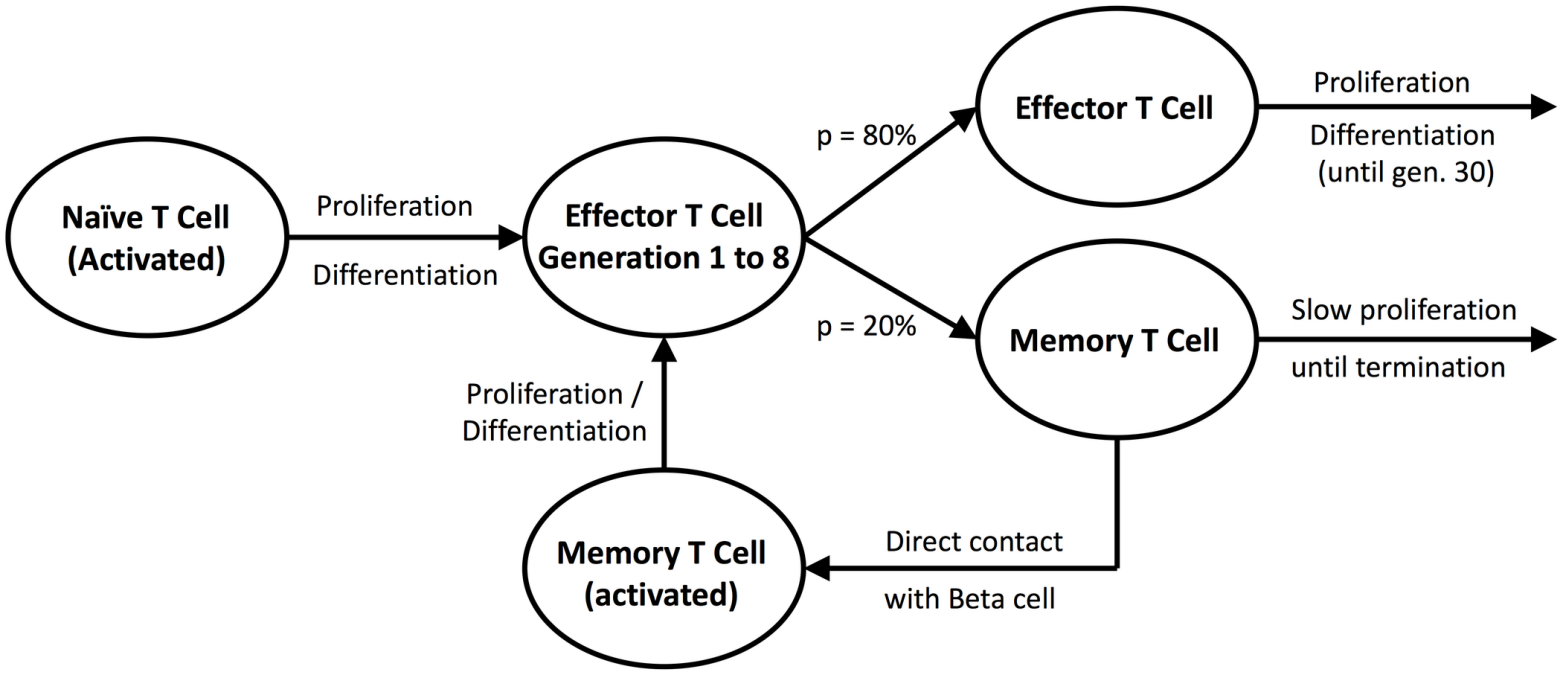
T cell proliferation process in the model. Here, p denotes percent probability. Rules were adapted from Kinjyo et al. [48].

T cell proliferation rules were implemented based on the findings of Kinjyo et al. [48] (Fig 1). According to this study, naive T cells enter a fast cell cycle upon contact with cognate antigen (*i.e*. Beta cells). This event is followed by fast proliferation of the naive T cells and starting from the 8th generation, the progeny has a 20% probability of differentiating into a memory T cell. Memory T cells have two subpopulations consisting of effector memory T cells, which exert rapid effector function, and central memory T cells, which lack immediate effector function and requires re-stimulation [49]. Since effector memory T cells display a similar function as the effector T cells, the model considers only the central memory T cells under the memory T cell designation.

Overall, T cell behavior in the model can be summarized as below:

- All T cell types exhibit random walk
- Naive T cells enter the cell cycle shown in Fig 1 upon contact with Beta cells.
- Effector T cells form a conjugate with up to two Beta cells upon contact, and kill with a 55% probability
- Memory T cells acquire effector function upon contact with its cognate antigen, *i.e*. Beta cells

The effector T cells in the model can conjugate with up to two targets simultaneously, as previously suggested by other modeling [50, 51] and experimental [52] studies. This model rule allows the targeting of a Beta cell by multiple T cells. When a conjugate is formed between a Beta cell and an effector T cell, the target has 55% probability of being destroyed, which was set based on experimental studies of Wiedemann et al. [52]. Since the conjugate can be preserved for a much longer period (1 to 2 hours, taken as 90 minutes in the model) in contrast to the time it takes to kill the target cell (several minutes) [42, 52], target cell death was assumed to take place upon conjugate formation. Also, effector T cells are assumed to avoid target selection during the time spent in the conjugate form.

### Beta cells

Beta cells trigger activation in naive T cells, and they can be recognized by all subsets of the T cell population in the simulation. The diameter of Beta cells was set as 10 *μm* [53] and a single cell was assumed to occupy a single grid (*i.e*. 10 *μm* × 10 *μm*). Also, insulin secretion from Beta cells was not considered.

Lately, there have been studies that suggest the presence of a residual pool of Beta cells in people with long-standing T1D, concomitant with the continual regeneration and subsequent destruction of Beta cells [31, 54–56]. Although Beta cell regeneration during T1D is still controversial, modeling is a strong tool to test this hypothesis, and therefore a parameter for Beta cell regeneration was incorporated into the model. Depending on the value of this parameter, Beta cells can start to proliferate at a set rate (as specified by the simulated scenario) upon encountering the autoimmune attack.

The rules for Beta cells can be summarized as follows:

- Proliferate at a set rate under immune attack (if Beta cell proliferation is enabled)
- Recognized by all T cell types and trigger proliferation or differentiation in T cells

### Basement membrane

Basement membrane is a key structure around the islets, which serves to prevent cell migration into the islet. Consequently, its destruction during the onset of the autoimmunity is a critical event that determines the fate of the islet. Some studies in the literature point towards reduced or delayed incidence of T1D through inhibition of enzymes such as heparanese, which degrade heparan sulfate, a key component of the basement membrane [57, 58].

Based on its role, the basement membranes for individual islets were also incorporated into the model. The basement membrane is represented by local values surrounding the islets, initially set to a predetermined value. This value is considered to be the same on all locations around the islet, initially. Over time, local values are gradually reduced by effector T cells, leading to the formation of openings in the basement membrane. Eventually, this allows the invasion of the islet by all T cell types and typically triggers a wave of proliferation in T cells. The representation of the basement membrane by an arbitrary and uniform initial value allows the calibration of the model against clinical data. Furthermore, this arrangement provides flexibility for simulating scenarios where basement membrane destruction is delayed through inhibition of degradative enzymes [57, 58]. Lastly, we do not consider the regeneration of the basement membrane as we assume the autoimmune response to be severe enough to prevent any regeneration.

Overall, the basement membrane is governed by the following rules:

- Constitute a barrier between all T cell types and the Beta cells
- Represented by local, arbitrary values around the islet, which are decreased by the effector T cells in the vicinity
- Repair of the membrane was not considered

## Results

Each scenario with a specific set of parameter values was analyzed by performing 100 repeated simulations with stochastic variations in T cell motility, cell cycle duration, and differentiation and target killing probabilities. The primary purpose here is to depict the capabilities of the model to replicate the innate behavior of the pancreatic tissue environment and the immune system components. At the same time, a potential target for the model is scenarios where this behavior is manipulated to evaluate the outcome of certain therapies, which may involve interventions such as autoimmune suppression and other therapies that may change the structural properties of the islets. Consequently, we have included a number of simulations where the system parameters are chosen above or below their expected values. Table 1 shows the model parameters, their units and the corresponding range considered in this study. Many parameter values were picked to represent either extreme of the potential values and mainly to verify that the model followed expected/reasonable trends. For Beta cell regeneration rate, we have observed that any value higher than 5% per day lead to an unrealistic replication behavior in the Beta cells (their population may increase to several times the initial value quickly. Such excessive replication is not reported in the literature). Also, values that were between 0 and 5% per day seemed not to impact simulation results as much to be included in the paper.

**Table 1:** Model parameters, units and the ranges considered in the simulations.

Model Parameter	Units	Range	Reference
Basement membrane strength	min area^−1^ cell^−1^	1440–20160	N/A (arbitrary value)
Islet density	% of tissue area	low (0.7–3.1) medium (2.9–5.2) high (4.2–7.7)	[59]
Islet diameter	*μm*	100–160	[53]
Initial T cell count	-	3–27	[38]
Beta cell regeneration rate	%	0–5	[60]

**Fig 2:**
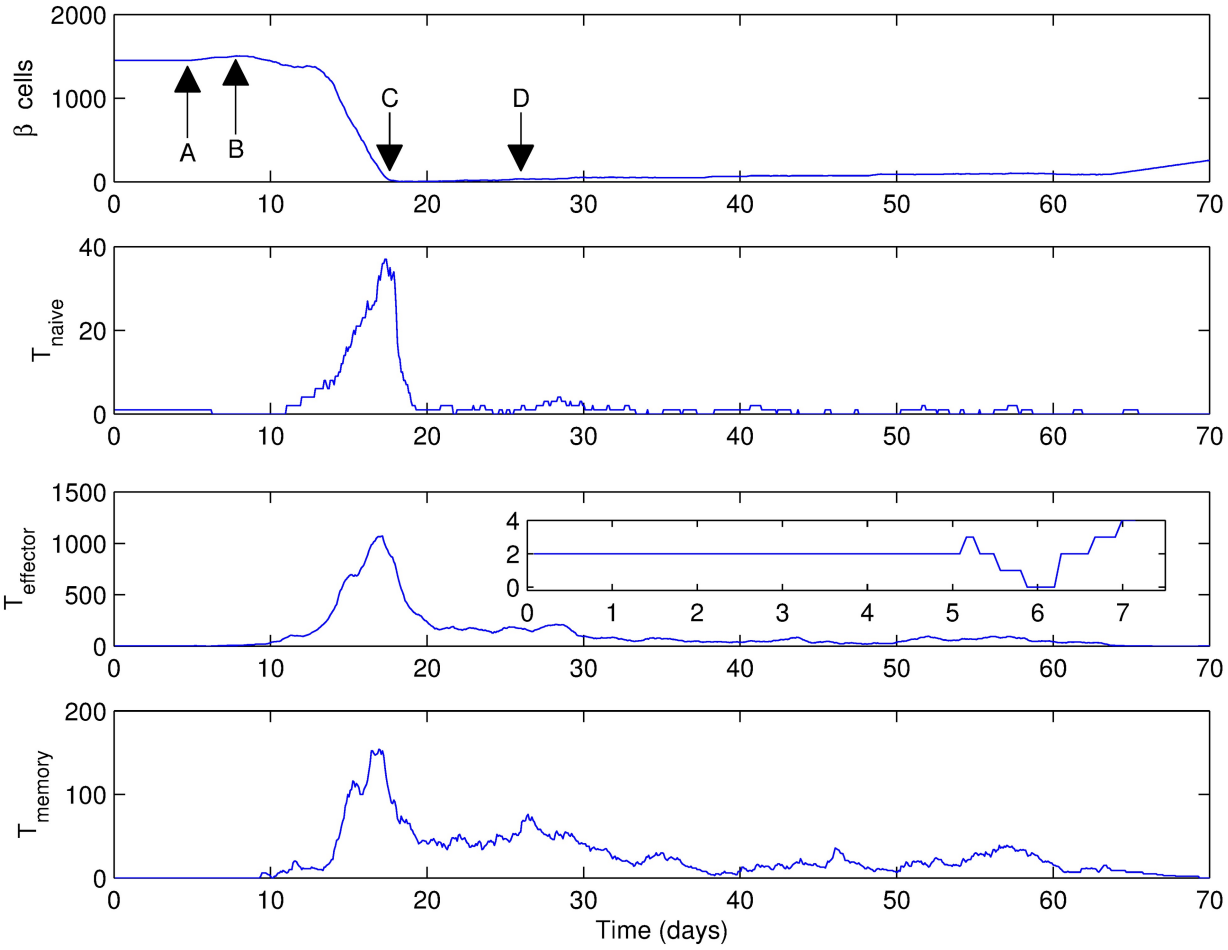
An example simulation output for a scenario where the basement membrane strength was set to 20160, Beta cell proliferation was 5% per day, islet density was medium and the initial T cell count was 3 with a 2:1 effector:naive T cell ratio. Labeled arrows show (A) t = 5 days (B) t = 8 days (C) t = 18 days (D) t = 26 days. Insets show the first 7 days. Note that t = 0 days corresponds to 4 weeks of age of the mouse.

**Fig 3:**
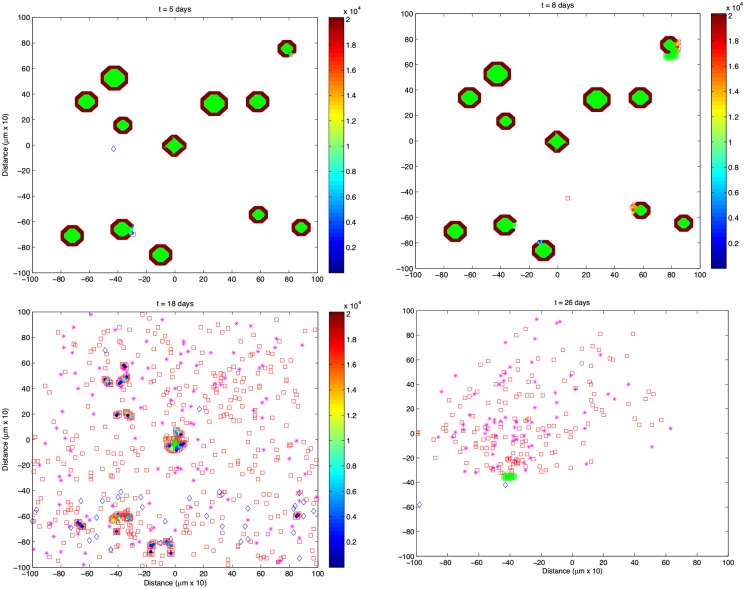
State of the simulation at the labeled arrows shown in Fig 2. Each tick on the x and y axes corresponds to 10 *μ*m. The basement membrane strength was set to 20160, Beta cell proliferation was 5% per day, islet density was medium and the initial T cell count was 3 with a 2:1 effector:naive T cell ratio. (A) t = 5 days, (B) t = 8 days (C) t = 18 days (D) t = 26 days. Note that t = 0 days corresponds to 4 weeks of age of the mouse. Color bar shows the intactness of the basement membrane. Beta cells are shown as green circles within the islets, while T cells can be seen as diamonds (◊, naive T cells), squares (□, effector T cells), and asterisks (*, memory T cells) outside the islets.

An example simulation output (Fig 2) shows the variation in the number of all cell types over a 10 week period. Snapshots of the simulation taken at points A, B, C and D on Fig 2 can be seen in Fig 3. In this example, basement membrane strength was set to 20160, the islet density was 4.1% (medium density), initial number of T cells was 3 with 2 effector and 1 naive T cell, and the Beta cell regeneration rate was 5% per day.

The layer around the islets represents the basement membrane, where the color indicates current intactness. Also, Beta cells are shown as green circles within the islets, while T cells are indicated as diamonds (◊, naive T cells), squares (□, effector T cells), and asterisks (*, memory T cells) outside the islets.

In Fig 2, no change in cell numbers is observed until day 5, which corresponds to the period of target search and dissolution of the basement membrane by the T cells. Starting from day 5, shown by point A in Fig 2 and in Fig 3a, an increase in Beta cells can be observed upon contact with the infiltrating effector T cells. Also, the number of effector T cells starts to increase in response to the increased Beta cell proliferation. This is essentially a consequence of increased probability of encountering a Beta cell in the vicinity due to the increasing population.

By day 8, the Beta cell population is reaching its peak (Fig 2 point B and Fig 3b), which has induced further increase in the T cell population. At this point, an islet that has grown over its original size due to regeneration appears in the upper right corner of Fig 3b. We also see the appearance of memory T cells in the tissue for the first time around day 9, which have a stable population until around day 13.

Following the increased Beta cell population, the T cell population has a dramatic increase after day 10, which leads to almost complete destruction of the islets by day 20 (Fig 2 point C and Fig 3c). At this point, the memory and naive T cell populations are at a considerable level, which mediate the rapid immune response toward the remaining islets. In Fig 3c, only the islet in the center remains along with residual small parts of other islets, which are completely engulfed by the T cells, and the Beta cells have been outnumbered. By day 20, all islet tissue has been destroyed except for a few (∼4) Beta cells and the T cells start to leave the tissue rapidly. This gives the few surviving Beta cells a window of opportunity for regeneration, which leads to the formation of a group of Beta cells at a very slow rate (Fig 2 point D and Fig 3d).

As a result of the randomized processes in the model, the time course of the events would show a certain amount of variability from one simulation to the other. Therefore, there would be cases where the complete destruction of the islets takes a longer or shorter period of time. Consequently, we have performed 100 simulations for any given scenario in order to make a judgement on the fate of the islets. For the repeated simulations, we have observed that the distribution of data at a given time point was far from normal. We use box and whisker plots to describe the dynamics of the autoimmune response. In the box plots given in the following sections, the top and the bottom of the boxes correspond to the third and first quartiles, respectively, whereas the median is marked by the circle on the boxes. The whiskers indicate the minimum and maximum cell counts that are not outliers for the given time point.

### Effect of basement membrane strength

**Fig 4:**
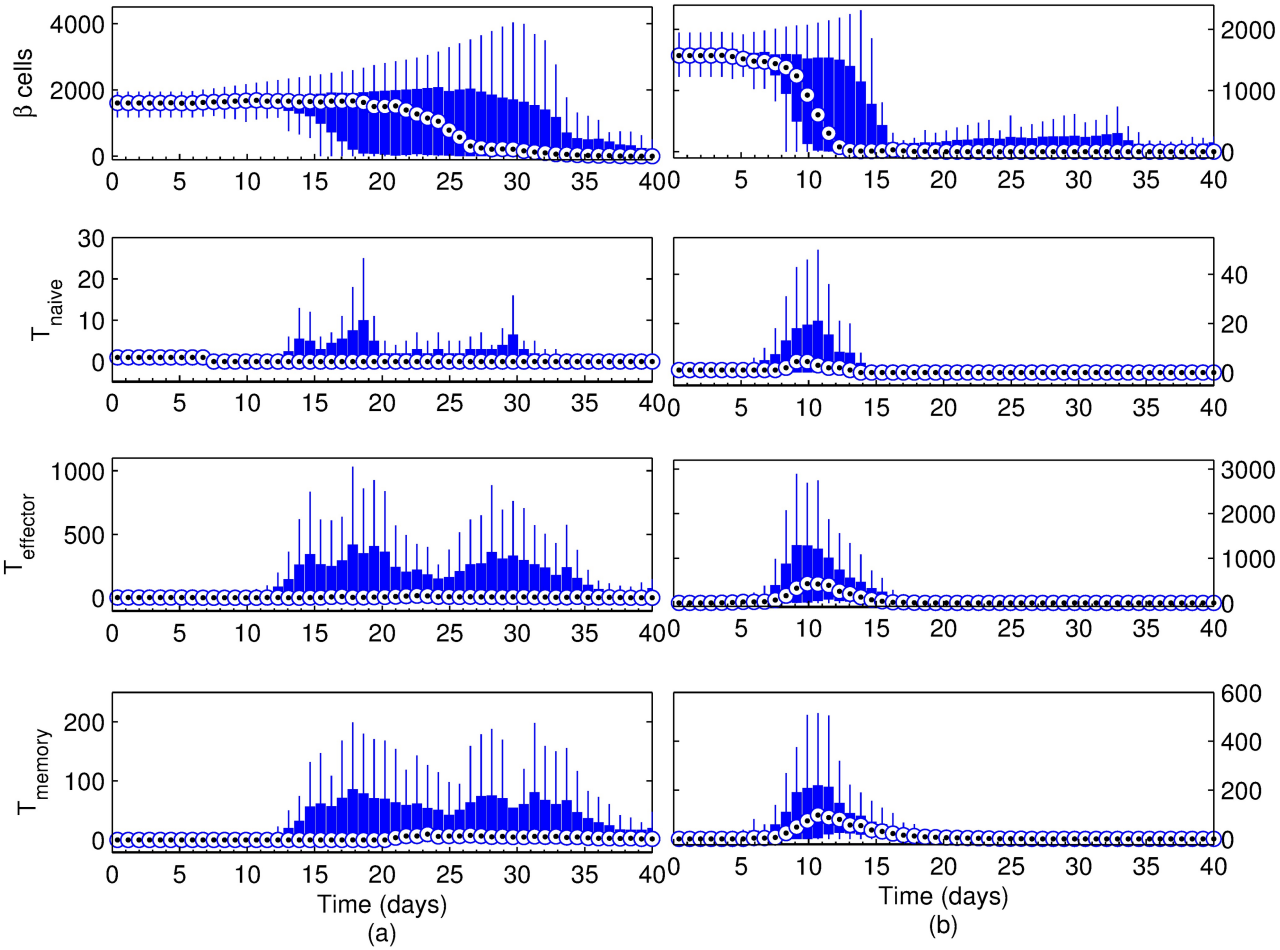
Simulation results for the scenario with a basement membrane strength of (a) 20160 and (b) 10080. Beta cell proliferation was 5% per day, islet density was medium and the initial T cell count was 3 with a 2:1 effector:naive T cell ratio. Changes after 40 days were not significant and hence not included (see S5 and S6 Figs for full figures.). Note that t = 0 days corresponds to 4 weeks of age of the mouse.

In order to test the effect of basement membrane strength on the progression and intensity of the autoimmune attack, we have performed simulations with the basement membrane strength set to 1440, 10080 and 20160. The Beta cell regeneration was set at 5% per day, islet density was medium, and there were three T cells initially. Results for the scenario where basement membrane strength was set to 20160 are shown in Fig 4a for the hundred simulations performed, including the one presented in Figs 2 and 3. In this case, the median Beta cell count barely increases in the first 15 days, while there is an increase in the maximum Beta cell count that indicates increased regeneration. Starting around day 12, however, the number of simulations with decreased Beta cell counts are more common, despite the stable median. This trend continues until day 20, after which the median Beta cell count also starts decreasing.

As expected, the T cell response coincides with the time period where Beta cell mass changes, and two distinct peaks emerge in T cell counts, which is most obvious in effector and memory T cell populations. Overall, the destruction of the islets occurs within a 2-week period, most likely to be between either the first and third weeks, or the third and sixth weeks.

**Fig 5:**
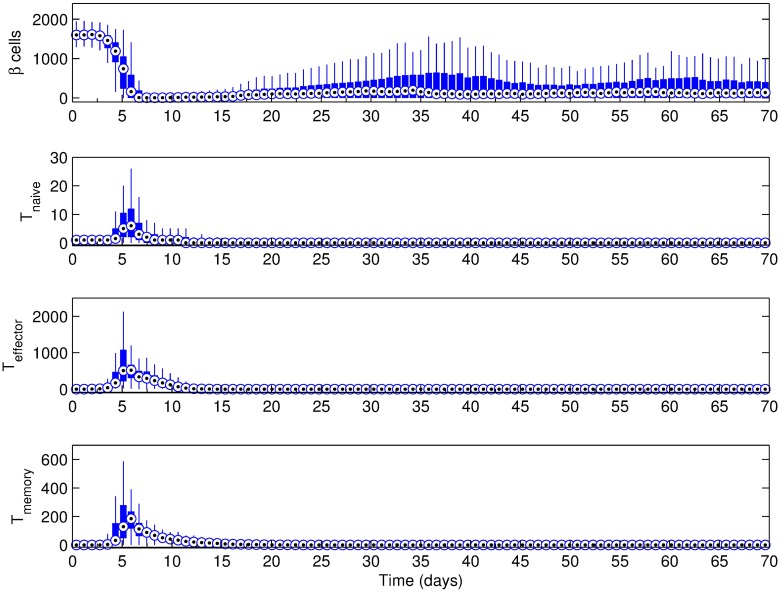
Simulation results for the scenario with a basement membrane strength of 1440. Beta cell proliferation was 5% per day, islet density was medium and the initial T cell count was 3 with a 2:1 effector:naive T cell ratio. Note that t = 0 days corresponds to 4 weeks of age of the mouse.

To further clarify the trends that relate to the basement membrane strength, scenarios where the basement membrane strength was set to 10080 and 1440 have been considered in Figs 4b and 5, respectively. By decreasing the basement membrane strength to half of its value in Fig 4a, the inflammation period is reduced to about a week (from day 6 to day 15) (Fig 4b). In addition, given the weaker basement membrane, the infiltration of the effector T cells can start as early as day 6. A notable difference in Fig 4b is in the T cell population. The peak values of all T cell types are much greater than what is observed in the stronger basement membrane scenario in Fig 4a. With further decrease of basement membrane strength from 10080 to 1440 (Fig 5), the onset of inflammation retires to the first few days of the simulation.

### Effect of beta cell regeneration

**Fig 6:**
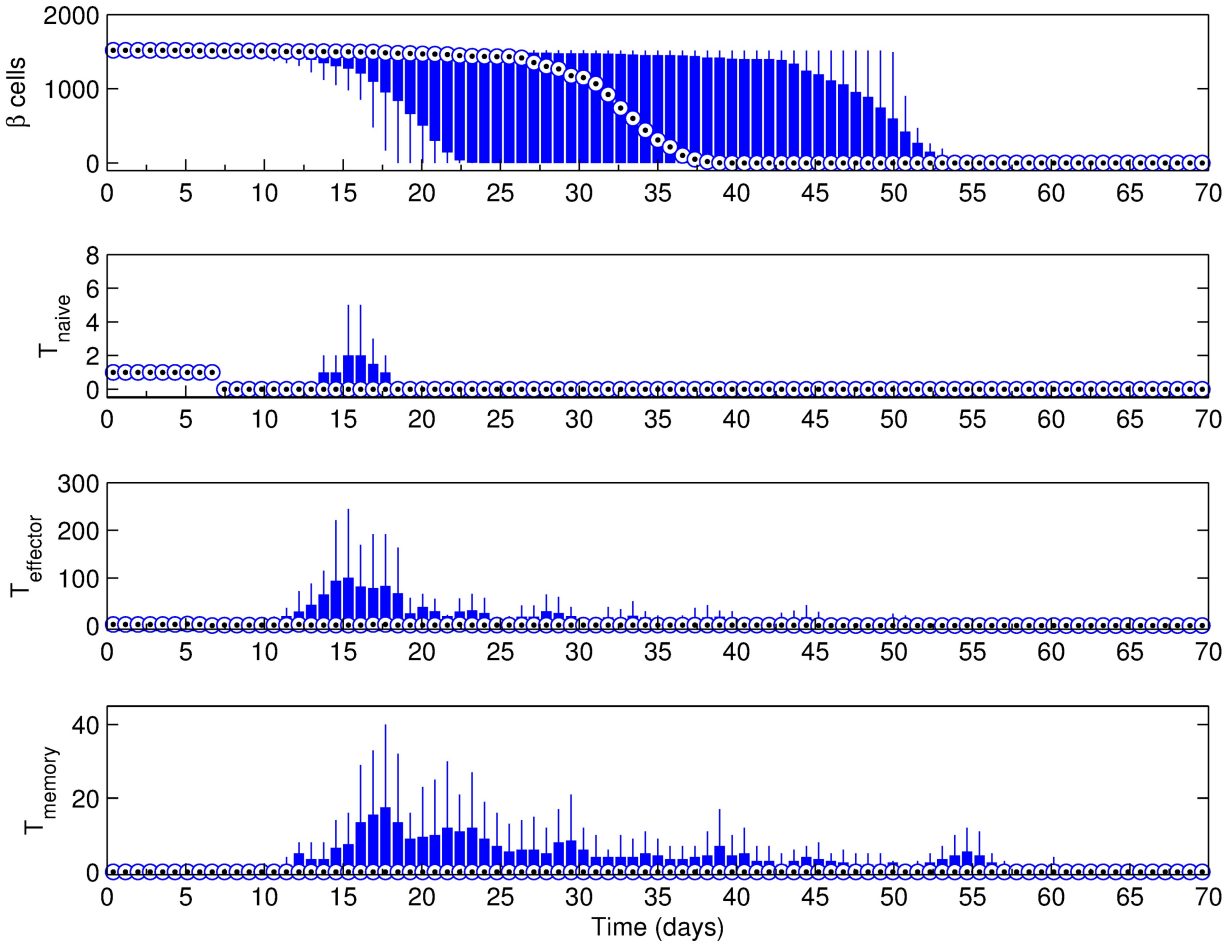
Simulation results for the scenario with a basement membrane strength of 20160. Beta cell regeneration was not allowed, islet density was medium and the initial T cell count was 3 with a 2:1 effector:naive T cell ratio. Note that t = 0 days corresponds to 4 weeks of age of the mouse.

The presence and extent of Beta cell regeneration is a current topic in diabetes research. In order to gauge the contribution of Beta cell regeneration to disease progression, we have conducted the same simulations reported in the previous section with no regenerative ability of the Beta cells. Fig 6 shows the simulation results for the scenario where the basement membrane strength was set to 20160. This scenario corresponds to the one in Fig 4a as all parameters were kept the same with the exception of Beta cell regeneration.

As compared to Fig 4a, the most striking feature of the scenario in Fig 6 is the broader window of islet destruction, which takes place between days 12 through 55. While the onset of inflammation is still on day 12 as in Fig 4a, the point where median initial Beta cell count is halved has shifted from day ∼23 in Fig 4a to day ∼32 in Fig 6. Also, the T cell response is several folds less severe than in Fig 4a.

Table 2 indicates that, as the basement membrane strength decreases, the role of Beta cell regeneration in the inflammation becomes less apparent. Although, there is a clear difference in the inflammation window as well as the time point where median Beta cell count is half of its initial value when the basement membrane is strong (BM = 20160), the difference is diminished at the moderate basement membrane strength (BM = 10080), and eventually disappears at very low basement membrane strength (BM = 1440).

**Table 2:** Comparison of the three basement membrane strength scenarios in the presence or absence of Beta cell regeneration. In all cases, the islet density was medium and the initial T cell count was 3 with a 2:1 effector:naive T cell ratio.

Basement membrane strength	Beta cell regeneration	Immune response	50% Beta cell loss
20160	Yes	days 12 ∼ 40	day 23
20160	No	days 12 ∼ 55	day 32
10080	Yes	days 5 ∼ 20	day 10
10080	No	days 5 ∼ 25	day 14
1440	Yes	days 0 ∼ 7	day 4
1440	No	days 0 ∼ 7	day 4

### Effect of initial T cell population

The simulation results given in previous sections have included an initial T cell population of 3, with a ratio of 2 effector T cells to 1 naive T cell based on the reported data in the literature [38]. However, the initial T cell number may be higher, especially in cases where the effector T cells cannot be contained sufficiently by the regulatory T cells [61]. Keeping the initial ratio of effector to naive T cells the same, we have performed simulations with 3, 9 and 27 initial T cells to observe the effect on the progression of autoimmunity. For these simulations the Beta cell regeneration rate was 5% per day, islet density was medium, and the basement membrane strength was set to 20160.

**Fig 7:**
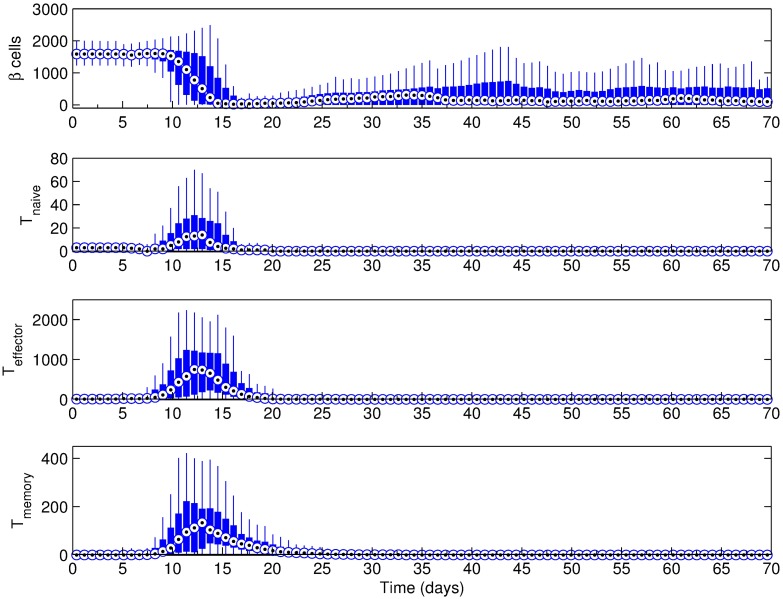
Simulation results for the scenario with a basement membrane strength of 20160. Beta cell proliferation was 5% per day, islet density was medium and the initial T cell count was 9 with a 2:1 effector:naive T cell ratio. Note that t = 0 days corresponds to 4 weeks of age of the mouse.

**Fig 8:**
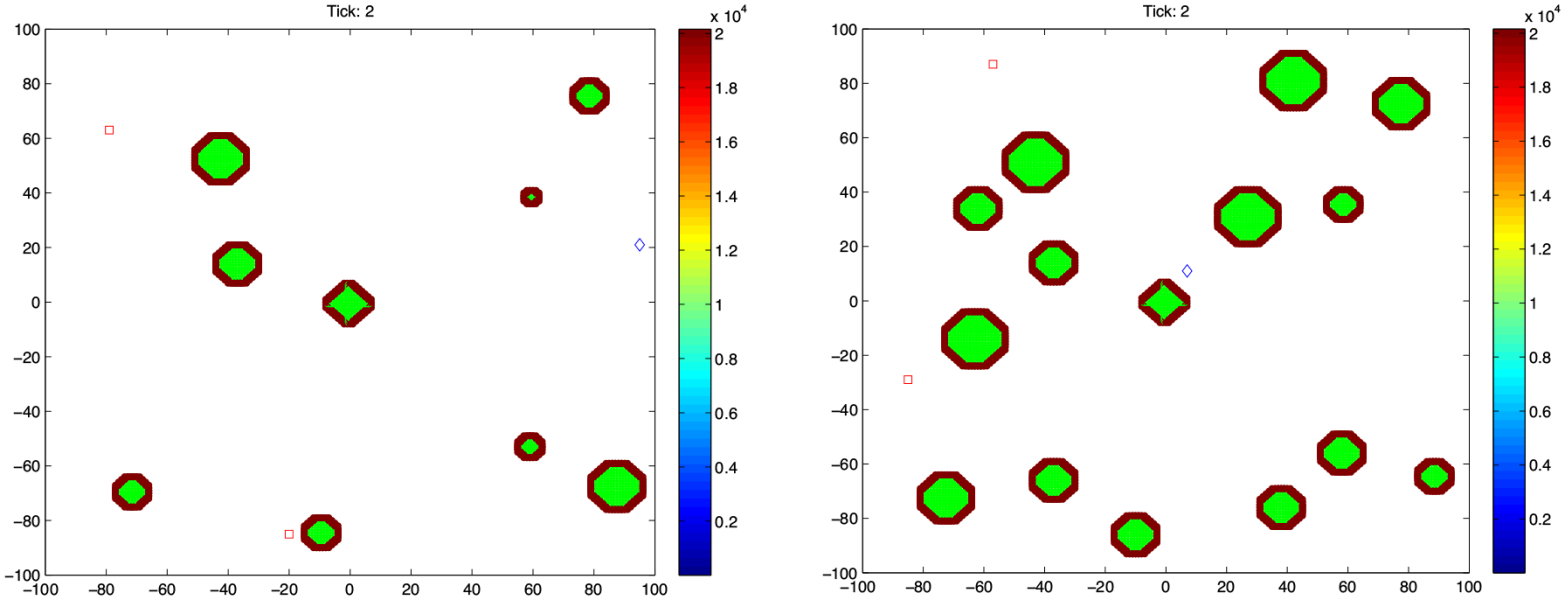
Examples snapshots of the simulations for (a) low (2.27% of tissue area, 9 islets) and (b) high (6.35% of tissue area with 15 islets) islet densities. Here, the basement membrane strength was set to 20160, Beta cell proliferation was 5% per day, and the initial T cell count was 3 with a 2:1 effector:naive T cell ratio. Note that t = 0 days corresponds to 4 weeks of age of the mouse. Color bar shows the intactness of the basement membrane. Beta cells are shown as green circles within the islets, while T cells can be seen as diamonds (◊, naive T cells), squares (□, effector T cells), and asterisks (*, memory T cells) outside the islets.

**Fig 9:**
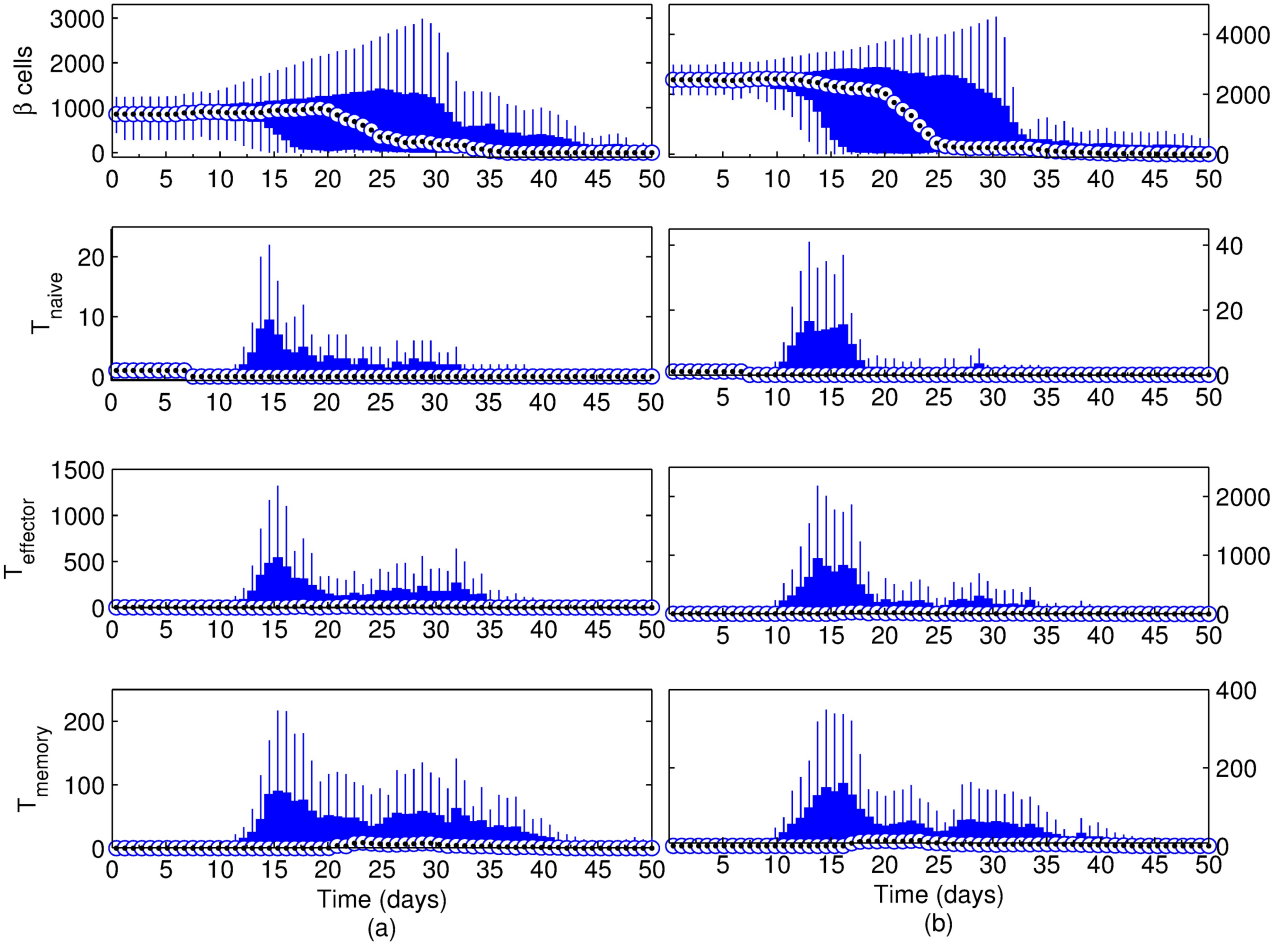
Simulation results for the scenario with a basement membrane strength of 20160. Beta cell regeneration was 5% per day, islet density was (a) low and (b) high; the initial T cell count was 3 with a 2:1 effector:naive T cell ratio. Changes after 50 days were not significant and hence not included (see S7 and S8 Figs for full figures.). Note that t = 0 days corresponds to 4 weeks of age of the mouse.

Despite the seemingly small increase in the initial T cell population from 3 (Fig 4a) to 9 (Fig 7), there is a dramatic reduction in the inflammation period. With the increase in initial T cell count, the onset of inflammation occurs earlier at around day 6 and lasts until around day 20. Additionally, the T cell response occurs within a shorter time period at a higher level in Fig 7. In this case, the median Beta cell count falls to 50% of its initial value around day 12, which is significantly lower than the case in Fig 4a (day 23). A further increase in the initial T cell population to 27 brings the first immune response time to an even earlier point at around day 4 (Table 3), which lasts until around day 14 (S4 Fig). In this case, the initial median Beta cell population is halved by day 8.

**Table 3:** Comparison of the different initial T cell count scenarios. In these simulations, the basement membrane strength was set to 20160, Beta cell regeneration was not allowed, and the islet density was medium.

Initital T cell count	Basement membrane strength	Immune response	50% Beta cell loss
3	20160	days 12 ∼ 40	23
9	20160	days 6 ∼ 20	12
27	20160	days 4 ∼ 14	8

### Effect of islet density

Earlier, we have shown that a larger Beta cell population has a higher chance of inducing a more severe immune response. Regardless of the presence or absence of Beta cell regeneration, one would expect a similar impact on disease progression at varying islet densities. Considering the variation of islet density between different parts of the pancreas, we have performed simulations at lower and higher islet densities, as denoted on Table 1. While some studies report islet density in terms of the number of islets within a certain cross-sectional area, [30, 62] others adhere to the islet (or Beta cell) area as a percentage of the investigated tissue area or both metrics [59]. We believe islet area as a percentage of the target tissue area is a more clear indicator of islet density, and therefore chose to report this metric for the following simulations.

Fig 8 shows examples of cases with low and high pancreatic islet density. An example of medium islet density was given in Fig 3a. In the low islet density example (Fig 9a), percentage of islet area was 2.27% (9 islets), whereas it was 4.10% (12 islets) and 6.35% (15 islets) for the medium (Fig 4a) and high (Fig 9b) density examples, respectively.

Despite the differences in the Beta cell population in each case, the window of immune response remains roughly the same in all three scenarios. The main noticeable difference is in T cell trends, which indicates that the peak inflammation is more likely to take place in the first few weeks of the simulation at higher islet densities. While there are two time periods where the inflammation is likely to happen in all three cases, the early immune response (days 10 ∼ 25) is clearly more severe than its late counterpart (days 25 ∼ 40) in the high islet density scenario.

## Discussion

The ABM developed is useful in investigating the potential approaches to delay the onset of T1D and in illustrating the effects of Beta cell regeneration. Simulation results indicate that the basement membrane can play a key role in delaying the onset of the autoimmune response. This suggests that therapies that focus on strengthening the basement membrane of the islets (*e.g*. heparanase inhibition [57, 58]) has the potential to lead to a smaller population of T cells in the pancreatic tissue, which can be exploited for T1D treatment.

As for the effect of Beta cell regeneration, it appears that the inflammation is much less severe and takes a longer time when there is no Beta cell regeneration (Fig 6). This may seem counterintuitive, as one would expect even less longevity from islets without regenerative ability. On the other hand, these findings are reasonable if one of the common features of autoimmune diseases is considered: epitope spreading.

Epitope spreading is the immune response that occurs towards an epitope which is different than the original one that initiated the immune response. While this is considered a protective feature of the immune system, it can contribute to the severity of the autoimmune diseases. Although the scale of our model does not cover antigens, the increase in Beta cell proliferation upon immune response increases the chances of unengaged T cells to contact and recognize other Beta cells. As the Beta cell count increases initially, the T cell population follows suit and leads to the severe inflammation observed in Fig 4a. In the absence of Beta cell regeneration, however, the likelihood of Beta cell contact decreases monotonically over time as Beta cell count decreases, which leads many of the T cells to migrate to neighboring tissues (*i.e*. leave the simulation area) or ultimately apoptose. We believe the sequence of these events in the model is equivalent to the mechanism of epitope spreading through exposure of T cells to different epitopes, therefore allowing our model to predict the expected fate of the pancreatic tissue under the presence/absence of Beta cell regeneration. Some studies that consider Beta cell regeneration indicate epitope spreading as a potential mechanism which leads to the destruction of the islets [61, 63, 64]. A greater body of literature also indicates increased proliferation in response to autoimmune response, rather than increased insulin demand due to hyperglycemia (*e.g*. [60, 65, 66]), which further confirms the possible role of epitope spreading in accelerated autoimmunity. We show the simulation results for basement membrane strength values 10080 and 1440 in Table 2, which also provides a comparison against the counterparts of these simulations in the previous section. Results of these simulations can be found in S2 and S3 Figs.

Based on the comparison of Beta cell regeneration scenarios in Table 2, it seems that there is a delicate balance between the basement membrane strength and the Beta cell regeneration rate, which ultimately determines the outcome of a potential inflammation. In the healthy pancreas, this balance is considered to be maintained by regulatory T cells. However, insufficient function or amount of these regulatory T cells is considered a leading cause of T1D [67, 68]. In such a case, our model findings, if clinically confirmed, would indicate therapies aimed toward the improvement of basement membrane strength and control of Beta cell regeneration alongside immunosuppression to maintain healthy islets in the pre-diabetic pancreas.

Whether there is increased regeneration of Beta cells or epitope spreading upon autoimmunity, the model lends itself as a tool for predicting the outcomes of different hypotheses qualitatively accurately. We believe such capabilities can be further improved with inclusion of more detailed rules and equations in the model, as well as higher resolution clinical data for the validation of the model parameters for quantitative predictions.

The more intense and quicker inflammation at higher initial T cell numbers (Fig 7) can be attributed to the increased probability of encountering a Beta cell due to the larger initial T cell population. This trend can be further confirmed at even higher initial T cell population scenarios (Table 3 and S4 Fig). Considering the many observations on NOD mice, which indicate a disease progression period of several weeks, it is reasonable to assume that the initial T cell population involved in the inflammation to be relatively small.

When the islet density in the scenario shown in Fig 4a is changed to low (Fig 9a) or high (Fig 9b), the two distinct peaks of inflammation we discussed previously is preserved. Among the two likely inflammation periods, the earlier one (days 10 ∼ 25) is clearly more severe than its late counterpart (days 25 ∼ 40) in the high islet density scenario. This trend can be explained by considering the larger and more abundant islets in the high density scenario, as shown in Fig 8b. Upon penetration of the basement membrane, the number of exposed Beta cells is greater than the low and medium density cases, which is likely to induce a more severe immune response early on. The fact that the window of immune response remains the same for different Beta cell counts further verifies that it is the regeneration of exposed Beta cells in response to an attack that drives the intensity and duration of the inflammation, rather than their initial population.

## Conclusion

We developed a model of the interactions between CD8^+^ T cells and Beta cells, which allows the observation of temporal variations in the cell populations, as well as the spatial interactions between individual cells. Beyond mimicking the clinical observation, the agent-based model has shown promise as a tool for testing various hypotheses *in silico*, providing capabilities to save time and resources for the experimental researchers, and facilitate knowledge discovery. The model predicted the emergence of a phenomena that is similar to epitope spreading, which illustrates an important advantage of ABM. Despite no explicit effort to include such a mechanism in the model, an ABM allows the emergence of certain phenomena that is analogous to the actual system under investigation. Modification of key model parameters may lead to the emergence of better outcomes from the *in silico* simulations, which would direct clinicians toward the design of the corresponding therapies. In the example of Beta cell regeneration and epitope spreading, this could indicate interventions involving immunosuppression along with the control of Beta cell proliferation.

## Supporting information

S1 Fig Summary of interactions between Beta cells and CD8^+^ T cells. (TIF)

S2 Fig Simulation results for the scenario with a basement membrane strength of 10080. Beta cell regeneration was not allowed, islet density was medium and the initial T cell count was 3 with a 2:1 effector:naive T cell ratio. Note that t = 0 days corresponds to 4 weeks of age of the mouse.
(TIF)

S3 Fig Simulation results for the scenario with a basement membrane strength of 1440. Beta cell regeneration was not allowed, islet density was medium and the initial T cell count was 3 with a 2:1 effector:naive T cell ratio. Note that t = 0 days corresponds to 4 weeks of age of the mouse.
(TIF)

S4 Fig Simulation results for the scenario with a basement membrane strength of 20160. Beta cell regeneration was 5% per day, islet density was medium and the initial T cell count was 27 with a 2:1 effector:naive T cell ratio. Note that t = 0 days corresponds to 4 weeks of age of the mouse.
(TIF)

S5 Fig Simulation results for the scenario with a basement membrane strength of 20160. Beta cell proliferation was 5% per day, islet density was medium and the initial T cell count was 3 with a 2:1 effector:naive T cell ratio. Note that t = 0 days corresponds to 4 weeks of age of the mouse.
(TIF)

S6 Fig Simulation results for the scenario with a basement membrane strength of 10080. Beta cell proliferation was 5% per day, islet density was medium and the initial T cell count was 3 with a 2:1 effector:naive T cell ratio. Note that t = 0 days corresponds to 4 weeks of age of the mouse.
(TIF)

S7 Fig Simulation results for the scenario with a basement membrane strength of 20160. Beta cell regeneration was 5% per day, islet density was low and the initial T cell count was 3 with a 2:1 effector:naive T cell ratio. Note that t = 0 days corresponds to 4 weeks of age of the mouse.
(TIF)

S8 Fig Simulation results for the scenario with a basement membrane strength of 20160. Beta cell regeneration was 5% per day, islet density was high and the initial T cell count was 3 with a 2:1 effector:naive T cell ratio. Note that t = 0 days corresponds to 4 weeks of age of the mouse.
(TIF)
