# Supplementary figures and images for: Agent-based modeling of the interaction between CD8+ T cells and Beta cells in type 1 diabetes
Source: PLoS One. 2018 Jan 10;13(1):e0190349. doi: 10.1371/journal.pone.0190349 (PMC5761894; doi:10.1371/journal.pone.0190349)

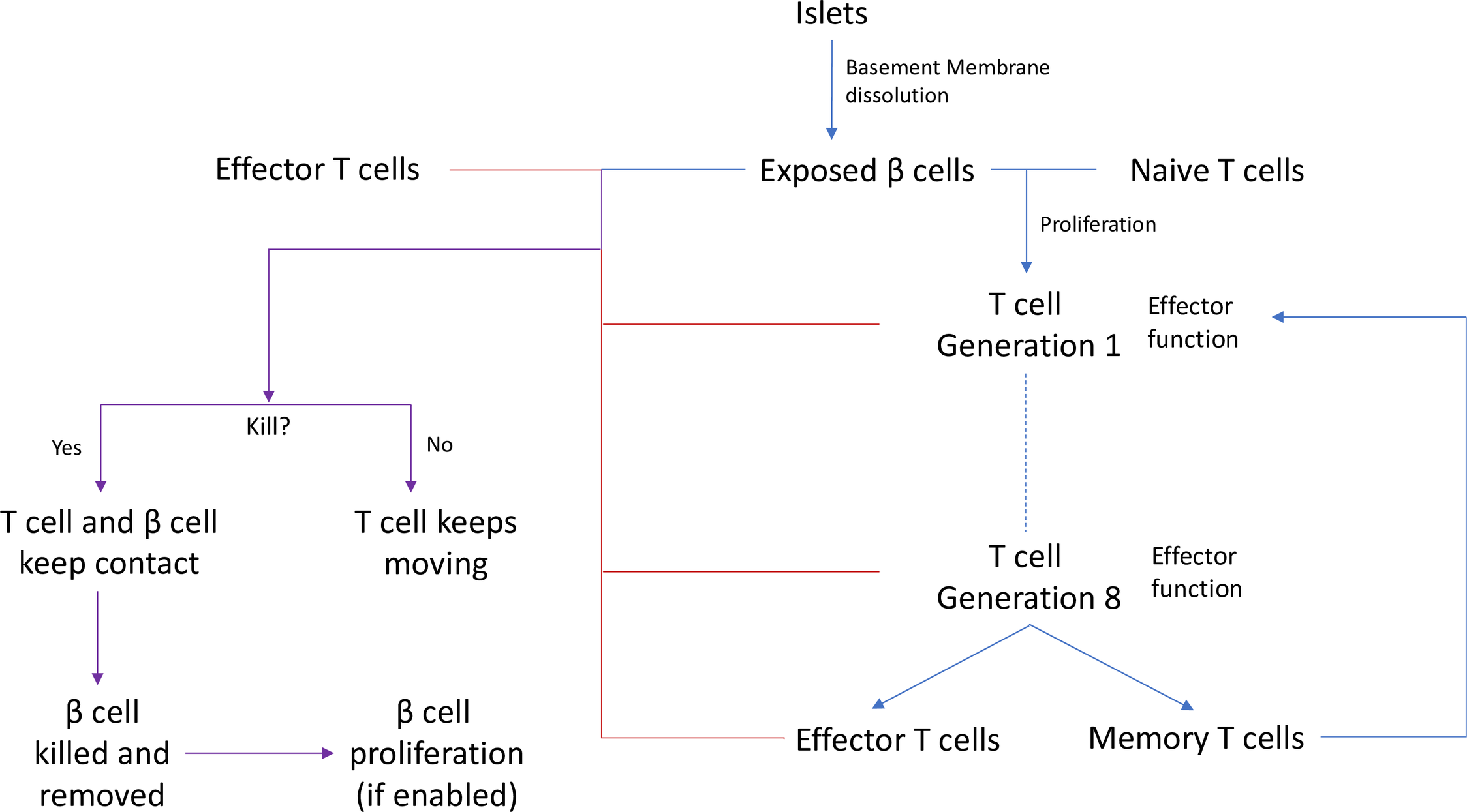

Supplement: S1 Fig — (TIF) [file pone.0190349.s001.tif]

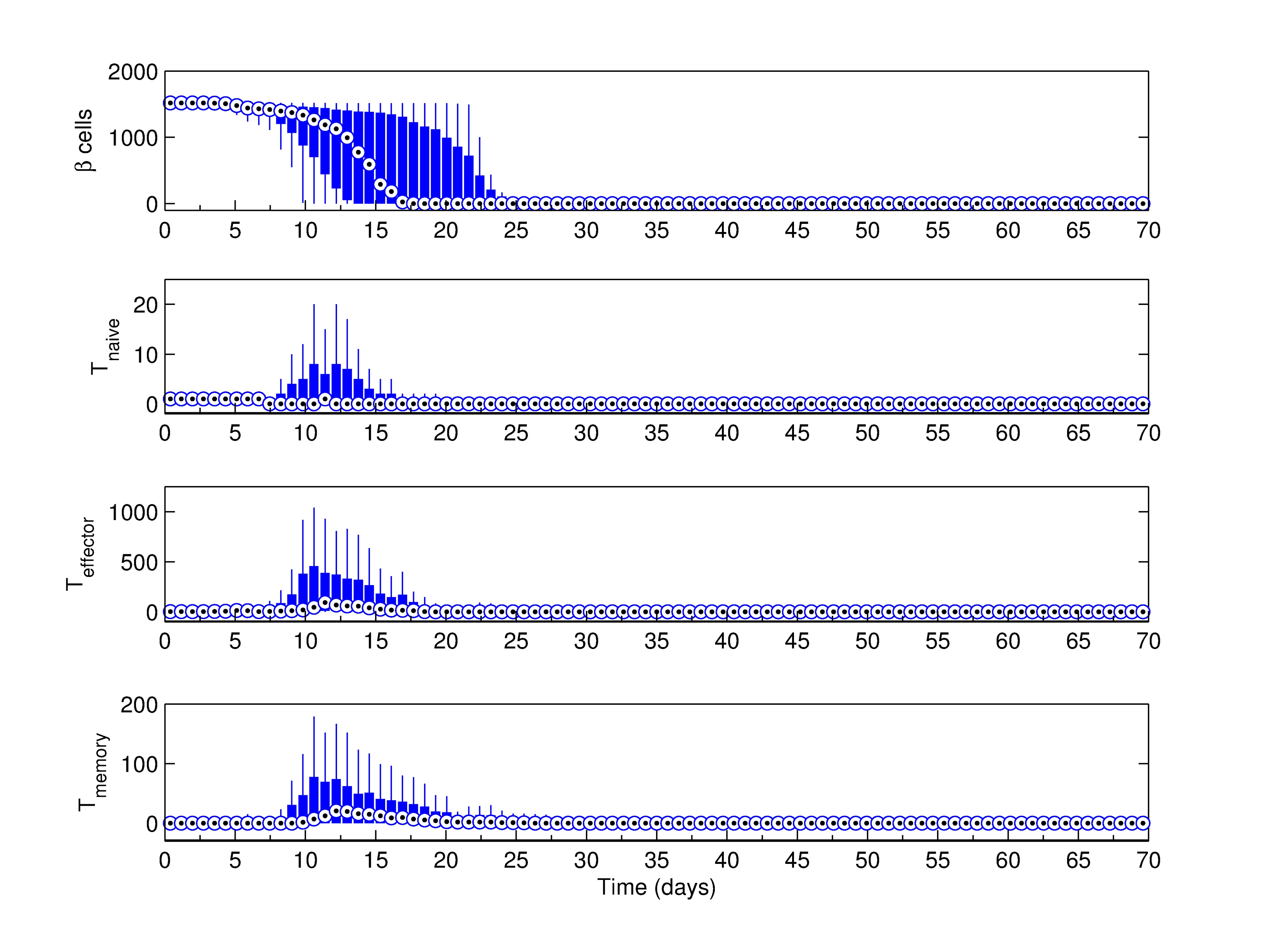

Supplement: S2 Fig — Beta cell regeneration was not allowed, islet density was medium and the initial T cell count was 3 with a 2:1 effector:naive T cell ratio. Note that t = 0 days corresponds to 4 weeks of age of the mouse. (TIF) [file pone.0190349.s002.tif]

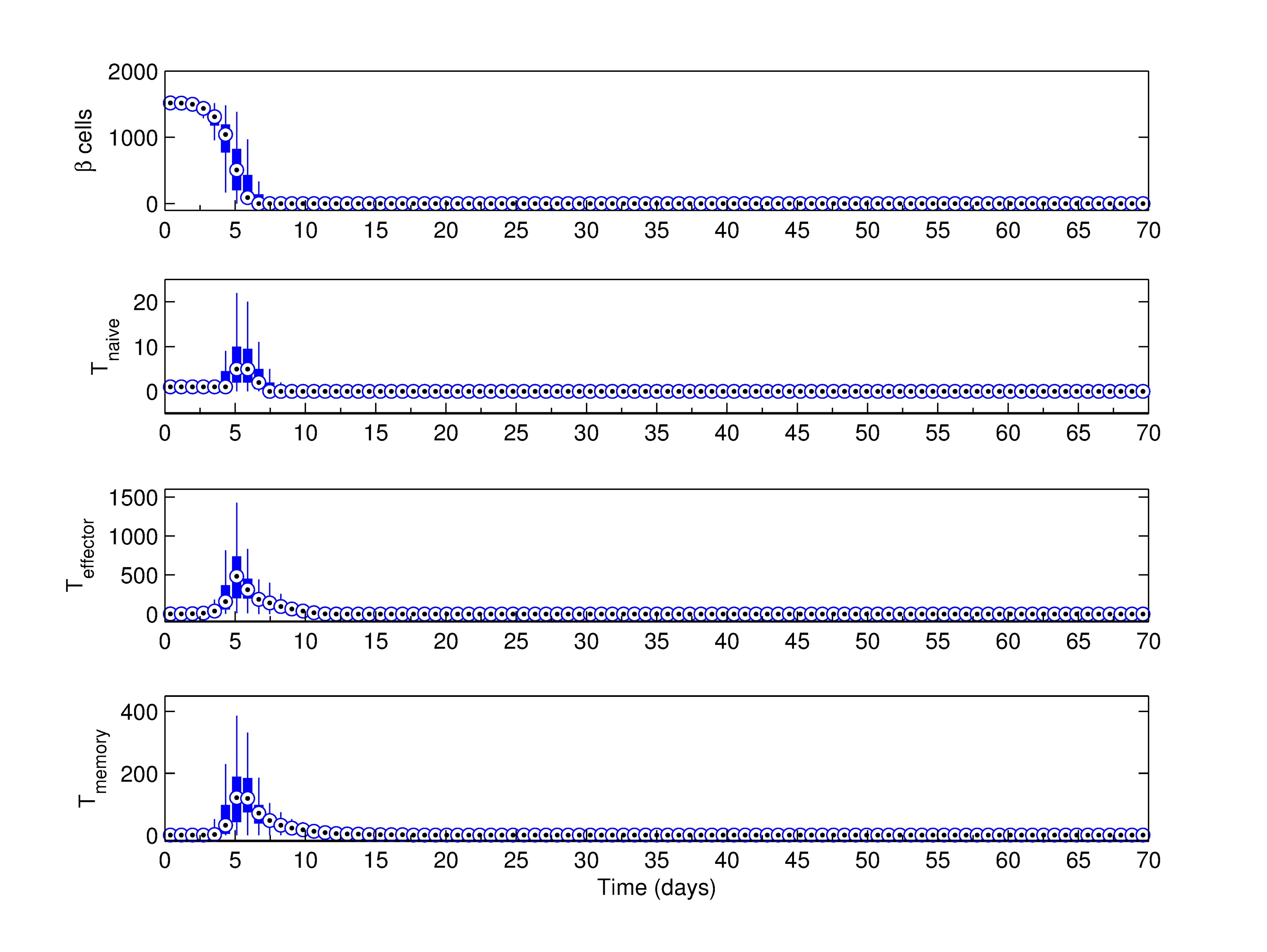

Supplement: S3 Fig — Beta cell regeneration was not allowed, islet density was medium and the initial T cell count was 3 with a 2:1 effector:naive T cell ratio. Note that t = 0 days corresponds to 4 weeks of age of the mouse. (TIF) [file pone.0190349.s003.tif]

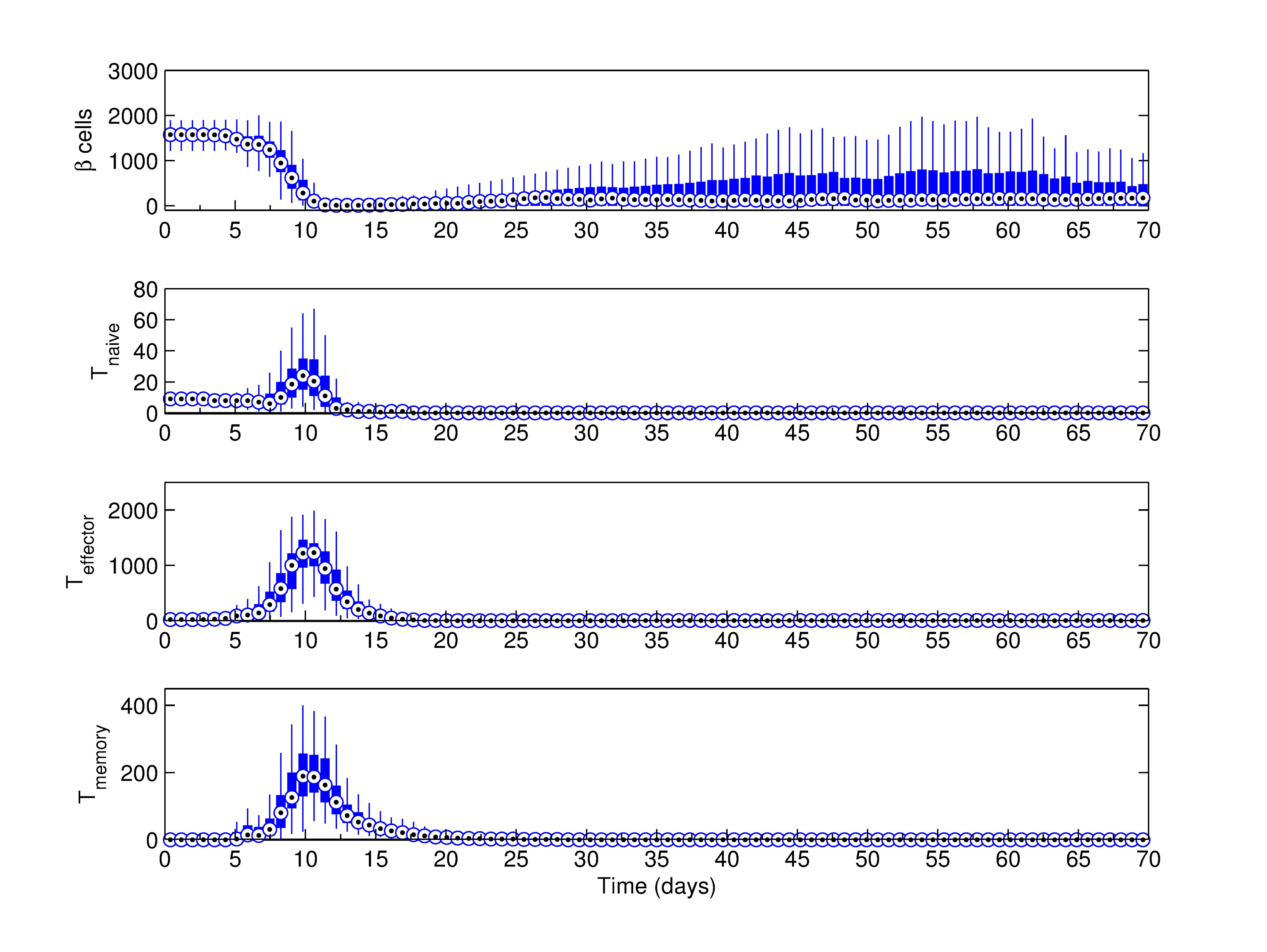

Supplement: S4 Fig — Beta cell regeneration was 5% per day, islet density was medium and the initial T cell count was 27 with a 2:1 effector:naive T cell ratio. Note that t = 0 days corresponds to 4 weeks of age of the mouse. (TIF) [file pone.0190349.s004.tif]

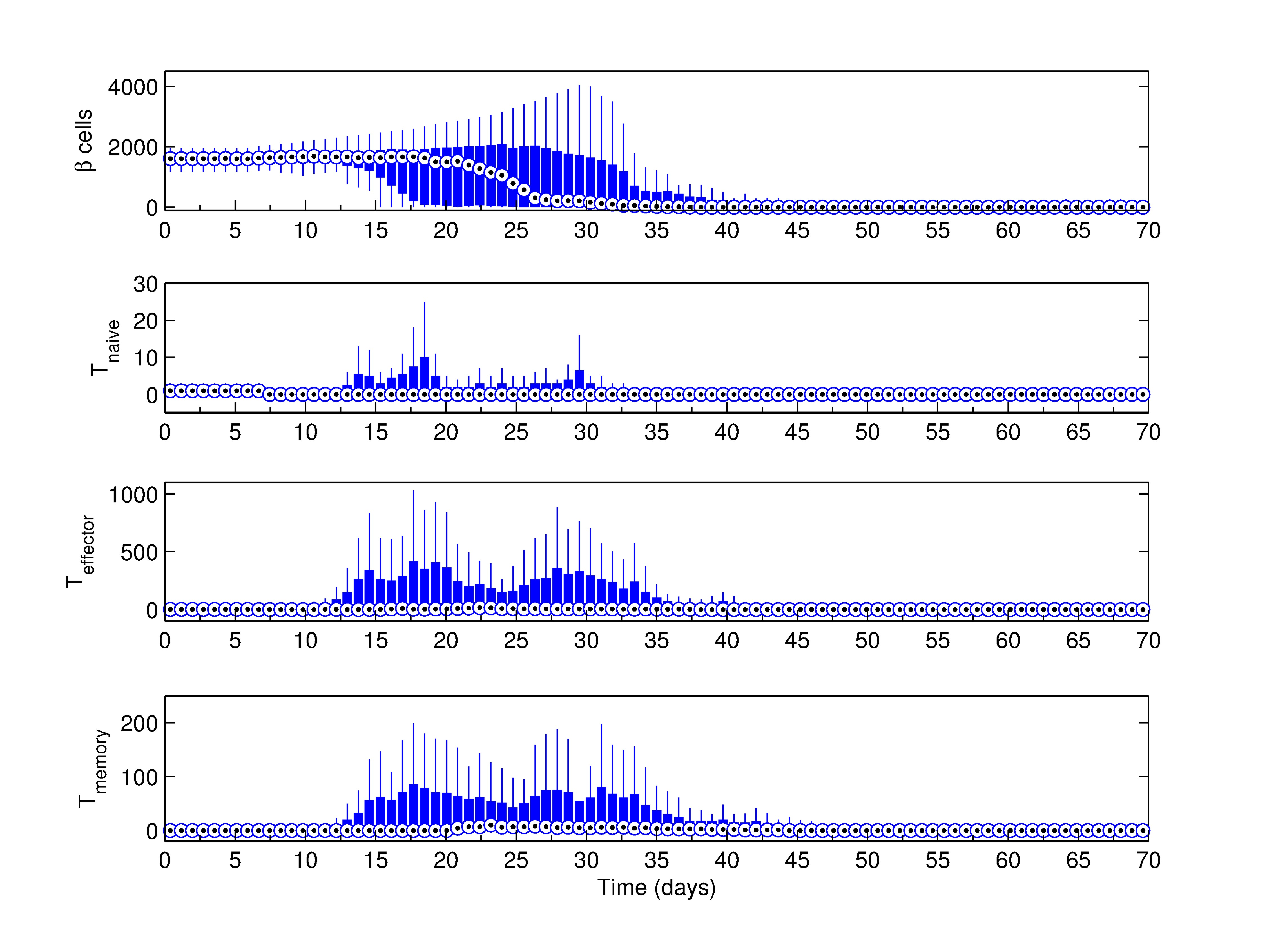

Supplement: S5 Fig — Beta cell proliferation was 5% per day, islet density was medium and the initial T cell count was 3 with a 2:1 effector:naive T cell ratio. Note that t = 0 days corresponds to 4 weeks of age of the mouse. (TIF) [file pone.0190349.s005.tif]

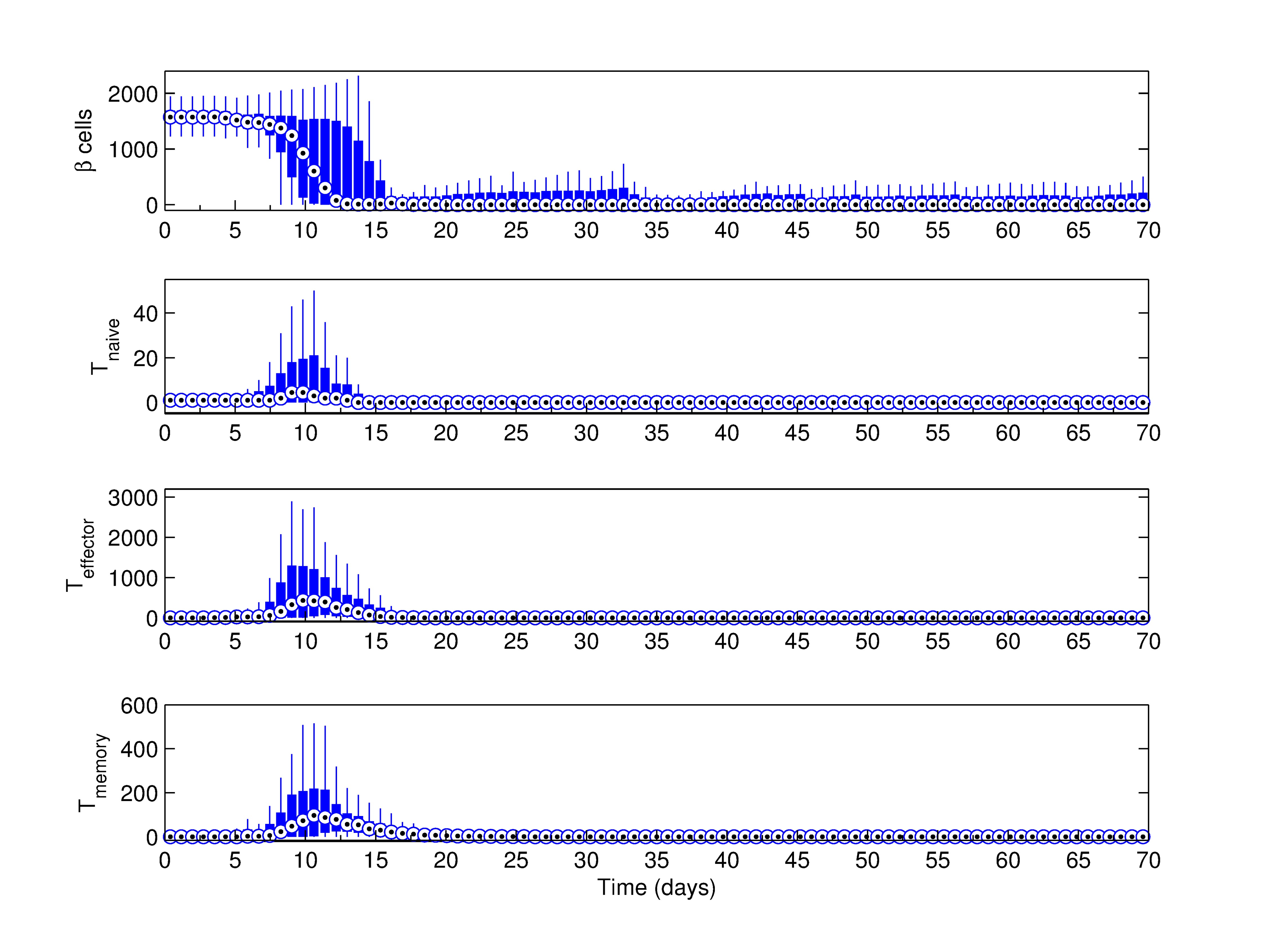

Supplement: S6 Fig — Beta cell proliferation was 5% per day, islet density was medium and the initial T cell count was 3 with a 2:1 effector:naive T cell ratio. Note that t = 0 days corresponds to 4 weeks of age of the mouse. (TIF) [file pone.0190349.s006.tif]

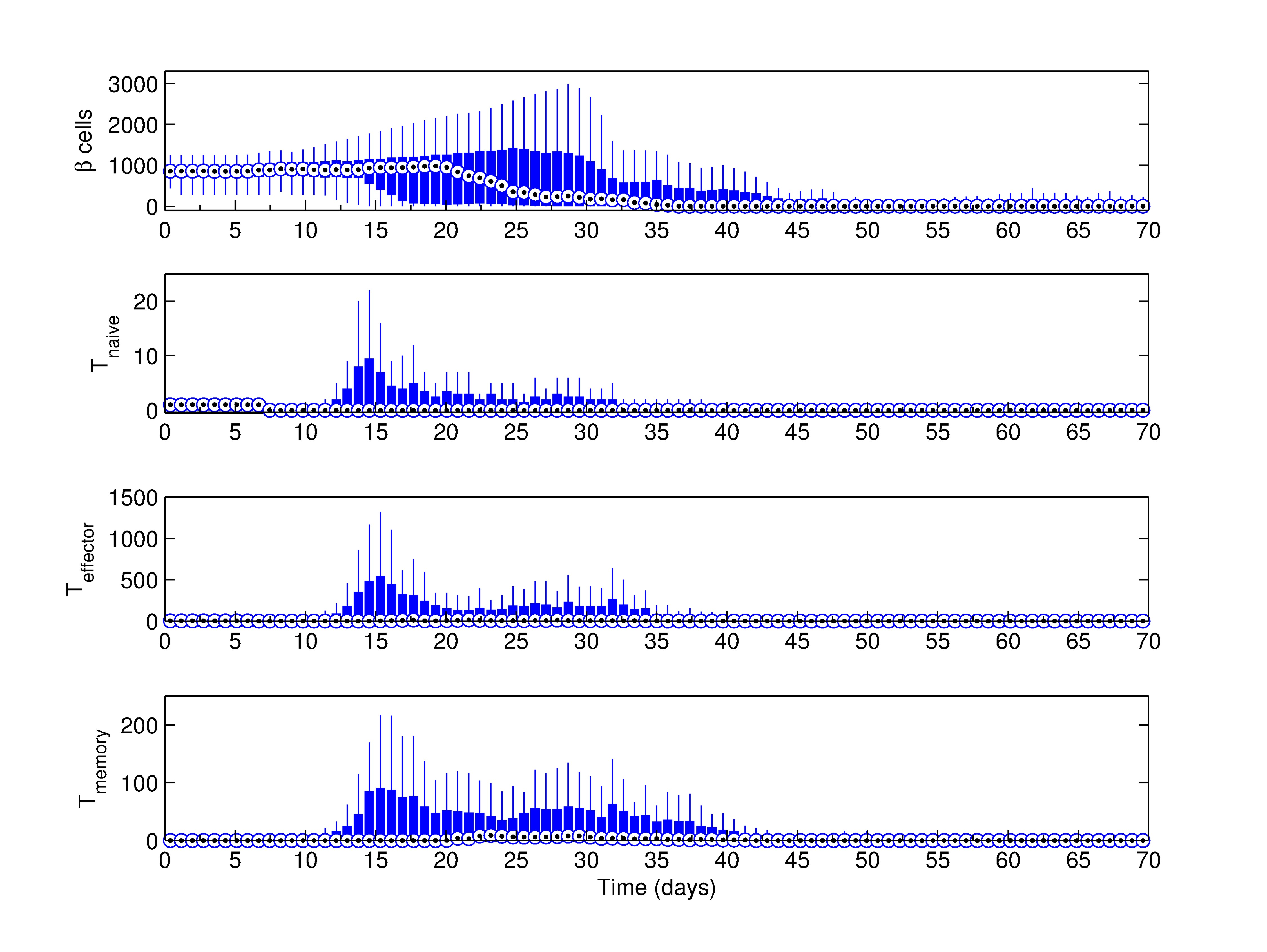

Supplement: S7 Fig — Beta cell regeneration was 5% per day, islet density was low and the initial T cell count was 3 with a 2:1 effector:naive T cell ratio. Note that t = 0 days corresponds to 4 weeks of age of the mouse. (TIF) [file pone.0190349.s007.tif]

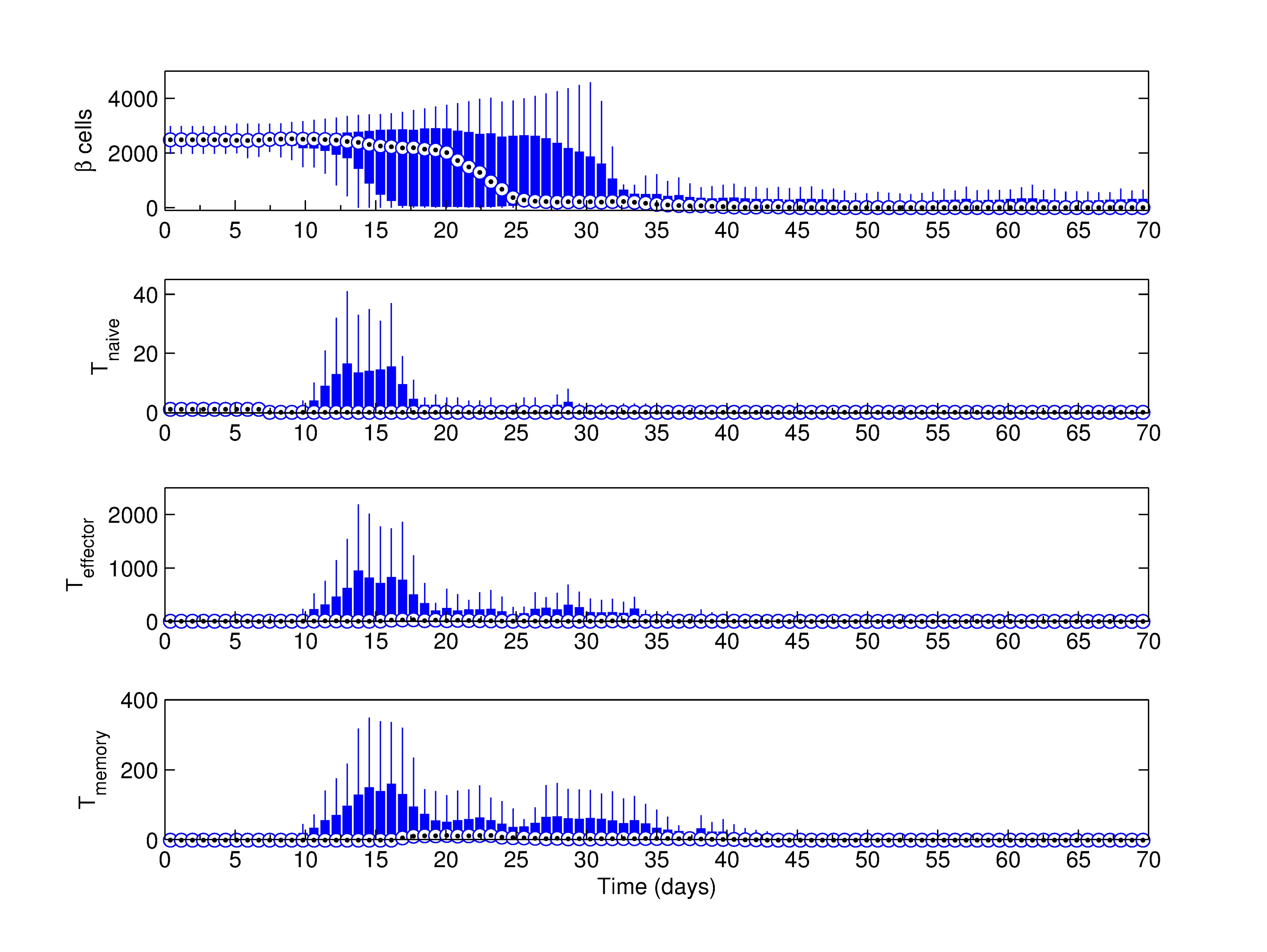

Supplement: S8 Fig — Beta cell regeneration was 5% per day, islet density was high and the initial T cell count was 3 with a 2:1 effector:naive T cell ratio. Note that t = 0 days corresponds to 4 weeks of age of the mouse. (TIF) [file pone.0190349.s008.tif]
